# Antibiotic Resistance of Commensal *Staphylococcus aureus* and Coagulase-Negative Staphylococci in an International Cohort of Surgeons: A Prospective Point-Prevalence Study

**DOI:** 10.1371/journal.pone.0148437

**Published:** 2016-02-03

**Authors:** Mario Morgenstern, Christoph Erichsen, Simon Hackl, Julia Mily, Matthias Militz, Jan Friederichs, Sven Hungerer, Volker Bühren, T. Fintan Moriarty, Virginia Post, R. Geoff Richards, Stephen L. Kates

**Affiliations:** 1 Department of Trauma Surgery, Trauma Center Murnau, Murnau, Germany; 2 AO Research Institute, Davos, Switzerland; 3 Virginia Commonwealth University School of Medicine, Richmond, Virginia, United States of America; 4 Paracelsus Medical University, Salzburg, Austria; Faculdade de Medicina de Lisboa, PORTUGAL

## Abstract

Nasal colonization with antibiotic resistant bacteria represents both a risk factor for the colonized individual and their immediate contacts. Despite the fact that healthcare workers such as orthopedic surgeons are at a critical interface between the healthcare environment and an at-risk patient population, the prevalence of antibiotic resistant bacteria within the surgical profession remains unclear. This study offers a snapshot of the rate of nasal colonization of orthopedic surgeons with multi-resistant staphylococci including methicillin-resistant *S*. *aureus* (MRSA) and methicillin-resistant coagulase-negative staphylococci (MRCoNS). We performed a prospective, observational study obtained at a single time point in late 2013. The participants were active orthopedic, spine and head & neck surgeons from 75 countries. The prevalence of nasal carriage of the different bacteria and the corresponding 95% confidence interval were calculated. From a cohort of 1,166 surgeons, we found an average *S*. *aureus* nasal colonization rate of 28.0% (CI 25.4;30.6) and MRSA rate of 2.0% (CI 1.3;2.9), although significant regional variations were observed. The highest rates of MRSA colonization were found in Asia (6.1%), Africa (5.1%) and Central America (4.8%). There was no MRSA carriage detected within our population of 79 surgeons working in North America, and a low (0.6%) MRSA rate in 657 surgeons working in Europe. High rates of MRCoNS nasal carriage were also observed (21.4% overall), with a similar geographic distribution. Recent use of systemic antibiotics was associated with higher rates of carriage of resistant staphylococci. In conclusion, orthopedic surgeons are colonized by *S*. *aureus* and MRSA at broadly equivalent rates to the general population. Crucially, geographic differences were observed, which may be partially accounted for by varying antimicrobial stewardship practices between the regions. The elevated rates of resistance within the coagulase-negative staphylococci are of concern, due to the increasing awareness of their importance in hospital acquired and device-associated infection.

## Introduction

*Staphylococcus aureus* and coagulase-negative staphylococci (CoNS) are both commensal and opportunistic pathogens, colonizing humans with an estimated prevalence of 30% and 100% respectively [1, 2]. These microorganisms are generally associated with asymptomatic skin and mucosal carriage, yet are paradoxically recognized as amongst the most frequent causative agents of hospital-associated infection (HAI) and device-associated infection (DAI) [3–5].

Antibiotic resistant strains, such as methicillin-resistant *S*. *aureus* (MRSA), have emerged as a significant threat in both the hospital and community environment [6]. Recent estimates suggest MRSA causes between 11,000 and 18,000 deaths, and 80,000 invasive infections in the US annually [4, 7]. With limited treatment options, MRSA infections are associated with higher mortality and increased financial costs [8, 9]. Methicillin resistance is also frequently observed within the CoNS (MRCoNS), particularly in surgical site infection (SSI) and DAI, where biofilm formation on implants and on tissue further reduces treatment success [10].

Identifying the source, reservoirs and vectors for the spread of antibiotic resistant bacteria poses significant challenges. The hospital environment, the patient's endogenous microflora, and health care workers (HCWs) may all play a role [11–14]. Evidence seems to indicate that the endogenous microflora of the patient may be critical, since clinical studies have found that *S*. *aureus* skin colonization increases the risk of a subsequent infection by three times and up to 80% of cases of staphylococcal bacteremia are caused by strains identical to those in the patient's nasal cavity [15, 16]. Furthermore, patient colonization with *S*. *aureus* is associated with a 2-9-fold increased risk of infection [6, 17]. Due to this apparent risk, routine screening of patients for colonization with *S*. *aureus* or MRSA upon hospital admission has been introduced into many healthcare settings including all Veterans Affairs hospitals in the US and numerous European countries [18, 19]. Such active surveillance programs have shown benefit in reducing outbreaks of nosocomial MRSA infections [20]. Interestingly, surgical patients have been shown to be at greater risk of developing HAI and surgeons themselves have even been shown, in small-scale studies, to be at an elevated risk of nasal carriage of MRSA in comparison with non-surgical medical doctors [7, 21]. HCWs are exposed to resistant bacteria in their workplace [22], which represents a risk to the HCW themselves, but also a potential factor in the spread of these bacteria within the hospital environment [23–27]. As such, the issue of screening HCWs for colonization with resistant bacteria has been broached, but the topic remains controversial [11, 23, 24, 28, 29]. Particularly since direct link between colonized HCWs and infection rate in their patients has not been established. The controversy centers upon the perceived sensitivity of the data, and the potential implications of colonization for the employer, the employee and the patient [24].

To explore the issue of colonization with antibiotic resistant bacteria within the surgical profession, we have prospectively evaluated the nasal carriage of staphylococci with their antibiotic resistance profiles in a large international cohort of active orthopedic, spine and head & neck surgeons. The primary aim of this study was to identify the prevalence of antibiotic resistant staphylococci present in the nares of active orthopedic surgeons. The secondary aim was to identify risk factors for nasal carriage of staphylococci based on personal and professional parameters.

## Materials and Methods

### Study design

In total 1,176 orthopedic, spine and head & neck surgeons from 75 different countries attending an international course in Davos, Switzerland, in late 2013 took part in this study. They represented 76% of the 1,200 participants and 350 faculty members present. Enrollment was voluntary, and the collected data was de-identified. All participants gave informed written consent and provided a nasal swab, alongside basic demographic and professional information. Bacterial identification and antibiotic susceptibility data were linked to the personal and professional information with a unique study participant code, which could not be linked with the individual participant in any way. Participants did not receive reimbursement for enrollment in the study.

### Surgeon screening and data acquisition

The questionnaire queried years practicing as a surgeon, location of place of work and birth (country and region), personal use of antibiotics within the past six months, treatment of patients with bone and joint infection, and treatment of patients with MRSA infection. No data was recorded on surgeon comorbidities or specific details of antibiotics taken. Data was entered via tablet computers into a REDCap electronic data capture tool, managed by our clinical research statisticians [30].

The surgeon participants circulated a dry swab (MASTASWAB^TM^, Mast Group Ltd., Italy) 5–6 times around the anterior nares of both nasal cavities and immediately placed it in the sterile swab container with protective Amies medium gel. Swabs were stored at 4–6°C at the end of each day and batches were transported to the clinical microbiology laboratory of the Trauma Center Murnau, Germany (maximum transportation time of four hours).

### Specimen processing and sample analysis

Bacterial culture and identification was performed according to the standard procedure for our hospital. In order to specifically culture Gram-positive bacteria, the swabs were streaked out on Gram-positive selective Columbia CNA (Colistin-Naladixic Acid) agar with 5% sheep blood (bioMérieux, Hazelwood, MO, USA) and subsequently incubated at 37°C for 48 hours. Morphologically distinct colony types with characteristic visual appearance of a staphylococcal species were identified and antibiotic susceptibility to 28 antibiotics was determined using a Vitek2 machine (bioMérieux Vitek Inc., Hazelwood, MO, USA). The antibiotic susceptibility pattern of all isolates was classified according to the European Centers for Disease Prevention and Control (ECDC) and the US Centers for Disease Control and Prevention (CDC) definition [31]. Using this definition bacteria were classified according to four different criteria: (1) oxacillin resistance; (2) total number of antibiotics to which the bacterium was resistant; (3) number of antibiotic classes to which the bacterium was resistant (out of 14 classes); and (4) multi-drug resistance. Oxacillin resistance is considered definitive for methicillin resistant status, and bacteria displaying oxacillin resistance will henceforth be described as MR (e.g. MRSA, MRSE, MRCoNS).

### Statistical analysis

Descriptive statistics were used to analyze surgeon characteristics and the antibiotic resistance patterns of the isolated bacteria. The prevalence of the different bacteria and the corresponding 95% confidence interval (95% CI) were calculated. Differences in the prevalence between subgroups of surgeons as well as differences in multi-drug resistance of bacteria were tested by the Chi-square test, or Fisher's exact test as indicated. Differences in the number of resistances (to 28 antibiotics) were tested by Kruskal-Wallis test. P-values <0.05 were considered significant. Statistical analyses were performed using SAS software (Version 9.2; Cary, NC, USA).

### Institutional Review Board approval

Institutional Review Board approval to perform this study was granted by the "Ethik-Kommission der Bayerischen Landesärztekammer", Mühlbauerstrasse 16, 81677 Munich, Germany (Approval number 13090).

## Results

### Participant demographics

Six of the 1,176 participants enrolled in the study were removed due to incomplete data and four were removed due to missing nasal swabs. Table 1 shows the demographic data of the remaining 1,166 included as study participants. Hospitals from 75 different countries were represented, with the most participants working in Switzerland (n = 178), followed by the Netherlands (n = 86), Germany (n = 56), USA (n = 53), Brazil (n = 50) and China (n = 47). Of the 95 countries of birth, the most participants were born in Germany (n = 101), followed by Switzerland (n = 86), the Netherlands (n = 83), USA (n = 57), Brazil (n = 49) and China (n = 48). The nationality, gender and age profiles were reflective of the course participant profile and included surgeons at all stages of their career.

**Table 1:** Demographic characteristics of participating surgeons.

Characteristics	n = 1166	n (%)
Age	younger than 36 years	422 (36.2)
	36–45 years	360 (30.9)
	46–55 years	272 (23.3)
	older than 55 years	112 (9.6)
Gender	Male	1026 (88.0)
	Female	140 (12.0)
Region of birth, N = 1164	Africa	58 (5.0)
	Asia	249 (21.4)
	Europe	624 (53.6)
	North America	78 (6.7)
	Central America and Caribbean	22 (1.9)
	South America	113 (9.7)
	Oceania	20 (1.7)
	Unknown	2 (0.2)
Region of work place, N = 1164	Africa	39 (3.4)
	Asia	231 (19.8)
	Europe	657 (56.4)
	North America	79 (6.8)
	Central America and Caribbean	21 (1.8)
	South America	108 (9.3)
	Oceania	29 (2.5)
	Unknown	2 (0.2)
Years practicing as a surgeon	less than 5 years	295 (25.3)
	5–10 years	292 (25.0)
	11–25 years	423 (36.3)
	more than 25 years	156 (13.4)
Type of hospital, N = 1154	Outpatient department	0 (0.0)
	Local hospital	334 (28.9)
	University hospital or level one trauma center	801 (69.4)
	Outpatient department + local hospital	4 (0.3)
	Outpatient department + university hospital or level one trauma center	3 (0.3)
	Local hospital + university hospital or level one Trauma center	8 (0.7)
	All three	4 (0.3)
	Unknown	12 (1.0)
Treated patients infected with MRSA within the last 6 months?	No	255 (21.9)
	Yes	911 (78.1)
Involved in treatment of bone or Implant infections within last 6 months?	No	165 (14.2)
	Yes	1001 (85.8)
Received antibiotic treatment within last 6 months?	No	909 (78.0)
	Yes	257 (22.0)

### Bacterial growth and antibiotic susceptibility

Overall, 95.3% of all swabs yielded Gram-positive bacterial growth. Two different species were cultured in 162 swabs (148 cases with one *S*. *aureus* and one CoNS, 14 cases with 2 different CoNS), resulting in a total number of 1,273 bacterial isolates from the 1,166 enrolled surgeons. The prevalence of each bacterial grouping is shown in Table 2.

**Table 2:** Prevalence of each bacterial species and grouping, n = 1166 surgeons.

Prevalence of nasal colonization*	n	% (95% CI)
No growth of any Gram-positive bacteria	55	4.7 (3.6;6.1)
*Staphylococcus aureus*	326	28.0 (25.4;30.6)
MRSA	23	2.0 (1.3;2.9)
MSSA	303	26.0 (23.5;28.6)
*Staphylococcus epidermidis*	591	50.7 (47.8;53.6)
MRSE	173	14.8 (12.8;17.0)
MSSE	418	35.8 (33.1;38.7)
Coagulase-negative staphylococci (CoNS)	933	80.0 (77.6;82.3)
MRCoNS* *	250	21.4 (19.1;23.9)
MSCoNS* *	681	58.4 (55.5;61.3)

* Prevalence in all 1166 surgeons

** Individual surgeons are included in both MRCoNS and MSCoNS categories, if both microorganisms were cultured from the same swab (n = 2). Four surgeons with a CoNS and missing information on oxacillin resistance were excluded.

Other than *S*. *epidermidis*, the CoNS species detected were: *Staphylococcus auricularis* (n = 8 surgeons, MR = 0%); *Staphylococcus capitis* (n = 12, MR = 0%); *Staphylococcus haemolyticus* (n = 46, MR = 28.9%); *Staphylococcus hominis* (n = 264, MR = 24.8%); *Staphylococcus intermedius* (n = 2, MR = 0%); *Staphylococcus lentus* (n = 4, MR = 0%); *Staphylococcus lugdunensis* (n = 12, MR = 0.1%); *Staphylococcus saprophyticus* (n = 1, MR = 0%); and *Staphylococcus warneri* (n = 7, MR = 0%).

**Fig 1:**
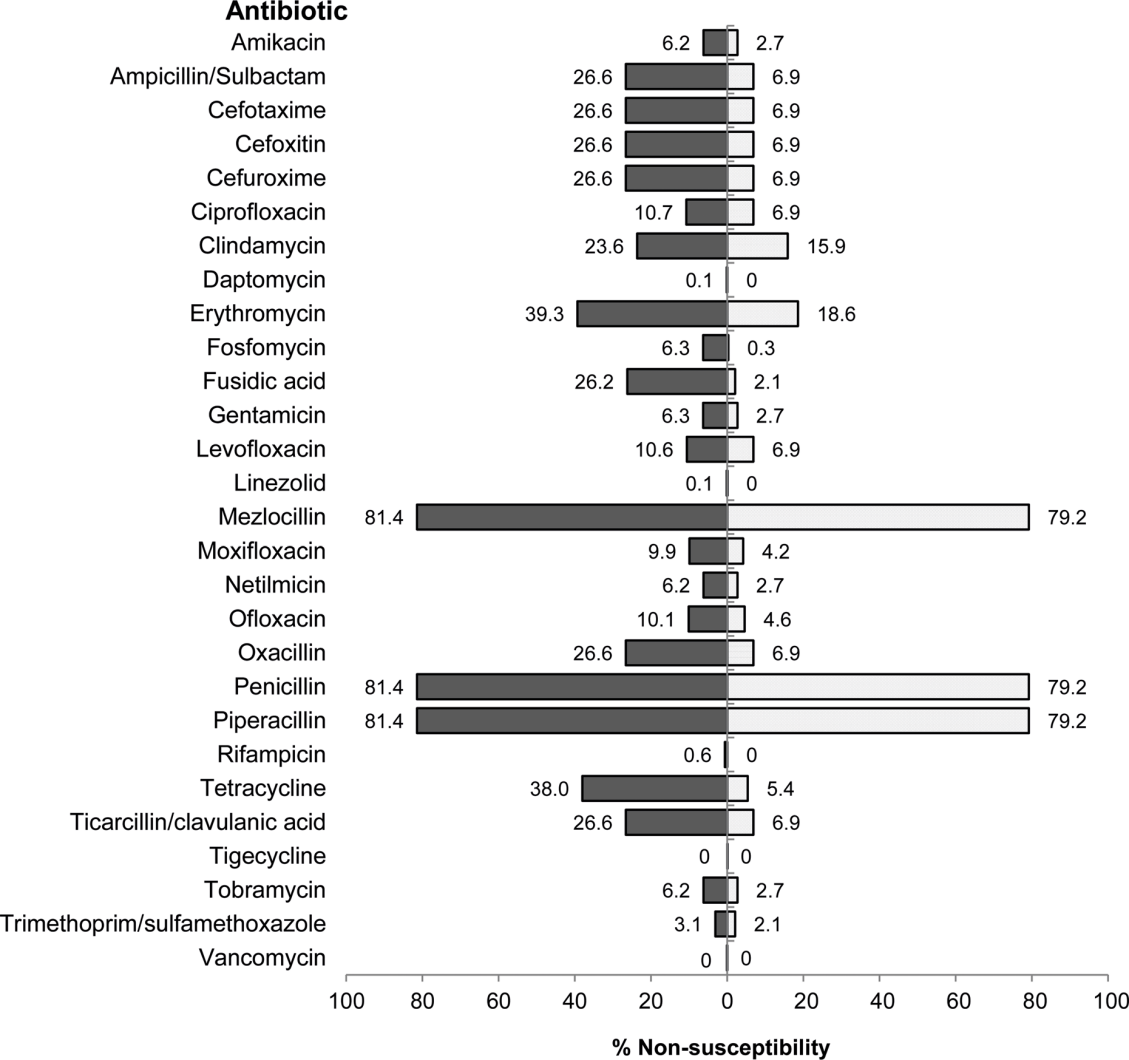
Non-Susceptibility of CoNS (left) and *S*. *aureus* (right), (%).

Antibiotic susceptibility testing was complete for all 28 antibiotics in 96.7% of isolates (Fig 1). Few notable resistance pattern were detected within the CoNS group, including strains resistant to rifampicin (1–≥32 μg/ml, n = 6), daptomycin (2 μg/ml, n = 1) and linezolid (≥8 μg/ml, n = 1), whereas Minimum Inhibitory Concentration (MIC) breakpoints defined by EUCAST were utilized [32, 33]. Multi-drug resistance (MDR), according to the ECDC definition [31], was present in 36.0% of all isolates (16.0% of *S*. *aureus*, 42.0% of *S*. *epidermidis* and 42.9% of all CoNS). No extensively-resistant or pan-drug-resistant isolates were identified [31]. The *S*. *aureus* isolates were, on average, non-susceptible (i.e. either intermediate or resistant) to 3.5 antibiotic agents, whilst for the entire CoNS group and the *S*. *epidermidis* isolates, it was 6.1 and 6.3 respectively. When grouped into classes, *S*. *aureus* isolates were, on average, completely resistant to 0.6 antibiotic classes, while CoNS were completely resistant to an average of 1.8 antibiotic classes.

### Risk factors for staphylococcal colonization

The prevalence of *S*. *aureus* was found to vary significantly between region of birth and region of work (Table 3). Within Europe, there was no significant intra-regional effect e.g. between Northern and Southern Europe, concerning *S*. *aureus* prevalence (Table 4). More than one fifth (22.0%) of the surgeon participants received systemic antibiotic treatment within the six months prior to the study (Table 1). Use of antibiotics did not result in an increase in culture negative swabs (p = 0.088), although significantly lower rates of nasal colonization with *S*. *aureus* (p = 0.043) were observed in these participants (Table 3). Nasal colonization with *S*. *aureus* was significantly lower in female (18.6%) than in male participants (29.2%, p = 0.008) (Table 3). No other factors were found to influence nasal colonization with *S*. *aureus*.

**Table 3:** Prevalence of *S*. *aureus*, MRSA and MRCoNS, n = 1166.

		*S*. *aureus*		MRSA		MRCoNS	
		n (%)	P value	n (%)	P value	n (%)	P value
**Region of birth, n = 1164**	Africa	8 (13.8)	**0.050**	2 (3.4)	**<0.001**	21 (36.2)	**<0.001**
	Asia	60 (24.1)		14 (5.6)		92 (37.1)	
	Europe	188 (30.1)		4 (0.6)		97 (15.6)	
	North America	17 (21.8)		0 (0.0)		9 (11.5)	
	Central America	7 (31.8)		1 (4.5)		7 (31.8)	
	South America	38 (33.6)		2 (1.8)		19 (17.0)	
	Oceania	6 (30.0)		0 (0.0)		5 (25.0)	
**Region of work place, n = 1164**	Africa	6 (15.4)	**0.041**	2 (5.1)	**<0.001**	19 (48.7)	**<0.001**
	Asia	55 (23.8)		14 (6.1)		92 (40.0)	
	Europe	191 (29.1)		4 (0.6)		99 (15.1)	
	North America	16 (20.3)		0 (0.0)		9 (11.4)	
	Central America	7 (33.3)		1 (4.8)		7 (33.3)	
	South America	40 (37.0)		2 (1.9)		17 (15.9)	
	Oceania	10 (34.5)		0 (0.0)		7 (24.1)	
**Received antibiotic treatment within last 6 months**	No	267 (29.4)	**0.043**	12 (1.3)	**0.003**	157 (17.3)	**<0.001**
	Yes	59 (23.0)		11 (4.3)		93 (36.3)	
**Age**	younger than 36 years	126 (29.9)	0.365	5 (1.2)	0.422	79 (18.7)	0.087
	36–45 years	105 (29.2)		8 (2.2)		78 (21.8)	
	46–55 years	69 (25.4)		8 (2.9)		72 (26.6)	
	older than 55 years	26 (23.2)		2 (1.8)		21 (18.8)	
**Gender**	Male	300 (29.2)	**0.008**	22 (2.1)	0.510	219 (21.4)	0.847
	Female	26 (18.6)		1 (0.7)		31 (22.1)	
**Years practicing as a surgeon**	less than 5 years	96 (32.5)	0.207	3 (1.0)	0.258	47 (15.9)	**0.027**
	5–10 years	75 (25.7)		4 (1.4)		70 (24.0)	
	11–25 years	116 (27.4)		11 (2.6)		103 (24.5)	
	more than 25 years	39 (25.0)		5 (3.2)		30 (19.4)	
Type of hospital*, n = 1135	Local hospital	101 (30.2)	0.180	5 (1.5)	0.414	76 (22.8)	0.541
	University hospital or level one trauma center	211 (26.3)		18 (2.2)		169 (21.2)	
**Treated patients infected with MRSA within the last 6 months?**	No	72 (28.2)	0.911	5 (2.0)	0.988	50 (19.7)	0.422
	Yes	254 (27.9)		18 (2.0)		200 (22.0)	
**Involved in treatment of bone or implant infections within last 6 months?**	No	37 (22.4)	0.087	1 (0.6)	0.234	35 (21.3)	0.954
	Yes	289 (28.9)		22 (2.2)		215 (21.5)	

* Only surgeons who work in one hospital type exclusively are considered

**Table 4:** Prevalence of *S*. *aureus* and MRCoNS within Europe (workplace), n = 657.

		*S*. *aureus*			MRCoNS*		
		No (%)	Yes (%)	P value	No (%)	Yes (%)	P value
**Region within Europe, n = 657**	Northern	71 (71.7)	28 (28.3)	0.151	93 (93.9)	6 (6.1)	**0.018**
	Eastern	37 (82.2)	8 (17.8)		33 (75.0)	11 (25.0)	
	Southern	70 (76.1)	22 (23.9)		77 (84.6)	14 (15.4)	
	Western	288 (68.4)	133 (31.6)		353 (83.8)	68 (16.2)	

* Missing data on methicillin resistance in 2 participants with CoNS

### Risk factors for antibiotic resistance

**Fig 2:**
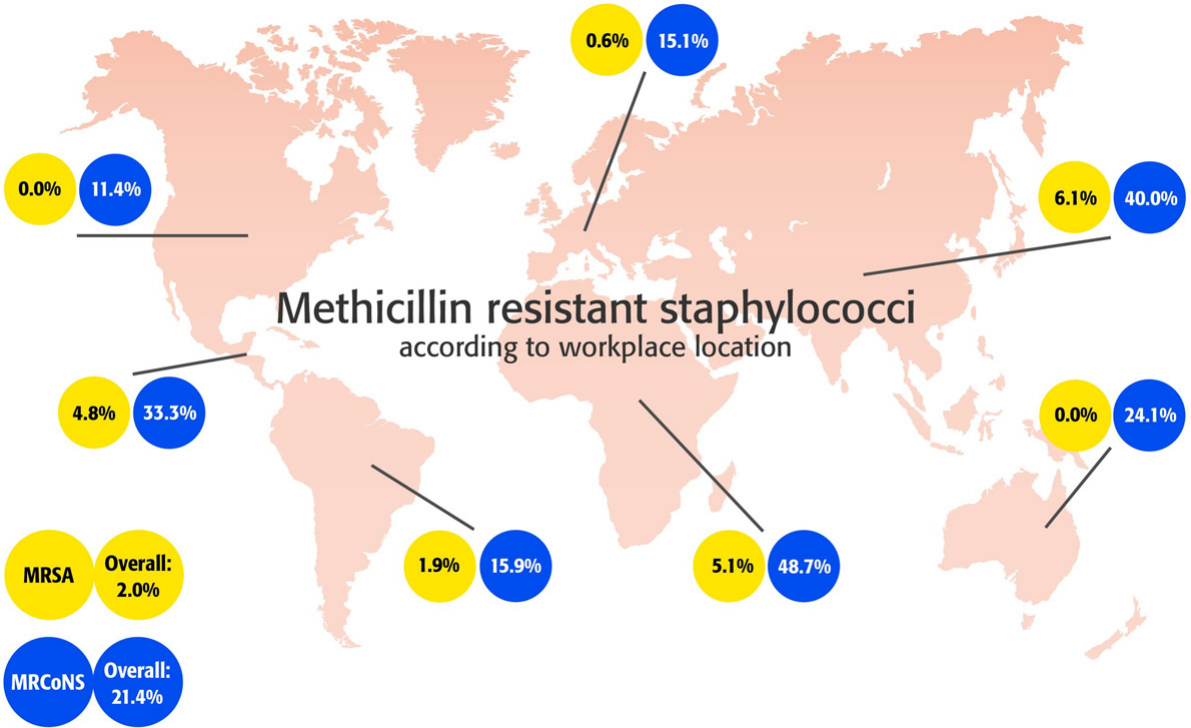
World map showing surgeon workplace and nasal carriage of MRSA and MRCoNS.

The prevalence of MRSA, MRCoNS, and specifically the prevalence of MRSE were found to vary significantly between region of birth and region of work (Tables 3 and 5, Fig 2). Similarly, MDR and the extent of resistances according to our four criteria were all affected by the region of birth and workplace for all bacteria (p<0.001). Within Europe, there was a significant intra-regional effect concerning MRCoNS incidence (Table 4). MRSA colonization was detected in just four participants from Europe, which is too few for inter-regional comparison.

**Table 5:** Participants with MRSA and MRCoNS by country of workplace.

Country of workplace*	Number of participants	Number with MRSA	Number with MRCoNS
Argentina	9	1	2
Australia	22	0	6
Austria	15	0	4
Belgium	33	1	5
Brazil	50	1	8
Canada	23	0	2
Chile	13	0	3
China	47	3	18
Colombia	19	0	0
Czech Republic	7	0	4
Denmark	30	1	1
Egypt	9	2	3
Estonia	7	0	2
Finland	12	0	1
Germany	56	0	17
Hungary	9	0	3
India	28	3	20
Indonesia	10	0	4
Ireland	9	0	4
Israel	16	0	4
Italy	38	0	5
Japan	18	2	5
Jordan	8	2	5
Lebanon	8	0	3
Mexico	16	1	5
The Netherlands	86	0	13
New Zealand	7	0	1
Norway	24	0	1
Peru	5	0	2
Poland	14	0	2
Russian Federation	11	0	1
Saudi Arabia	15	0	6
Singapore	15	2	3
Slovenia	21	0	5
South Africa	21	0	9
South Korea	5	1	2
Spain	21	1	2
Sri Lanka	5	1	4
Sweden	21	0	1
Switzerland	178	0	20
Thailand	13	0	2
Turkey	8	0	3
United Arab Emirates	5	0	0
United Kingdom	30	1	5
United States of America	53	0	7
Venezuela	5	0	1
Vietnam	5	0	4
Others**	86	0	22

*Only countries with at least 5 participants are listed

**Number of countries with less than 5 participants, n = 28

Antibiotic use did result in a significant increase in the prevalence of MRSA, MRSE and MRCoNS and the number of antibiotic non-susceptibilities (p<0.001). The MRSA prevalence in surgeons who had received antibiotic treatment was 4.3%, compared with 1.3% in those who had not received antibiotic treatment. The number of years of practice was found to influence the prevalence of MRCoNS and the extent of non-susceptibilities in CoNS (p = 0.022) but not methicillin resistance in *S*. *aureus*. However, no other factor was found to influence antibiotic resistance status of any staphylococcal species (Table 3).

## Discussion

The literature is lacking in an international and large-scale assessment of nasal colonization in the surgical profession. Against this background, we provide a global snapshot of nasal colonization with multi-resistant staphylococci in an international cohort of surgeons.

From a total population of 1,166 surgeons, we found an average *S*. *aureus* nasal colonization rate of 28.0% (CI 25.4;30.6), which is broadly comparable to previously described colonization rates in the general population: 27% in the UK and 29–32% in the US [34–36]. Studies investigating colonization of HCWs with *S*. *aureus* in regional cohorts from São Tomé and Príncipe, Iran, Norway and Thailand has also shown similar rates (20.2–29.7%) [37–40]. Some studies have indicated that HCWs may be colonized at a higher rate, whereby a *S*. *aureus* colonization rate of 21.7% was found in non- physicians and a rate of 37.4% in physicians [41], whereas in two West African hospitals, extensive colonization of both inpatients and HCWs by *S*. *aureus* (carriage rate 41%) was demonstrated [42]. Only a few studies have specifically analyzed *S*. *aureus* nasal colonization in surgeons [43]. Schwarzkopf *et al*. screened 135 orthopedic surgeons at the New York University Hospital for Joint Diseases and identified a rather high *S*. *aureus* colonization rate of 35.7% [43]. Our study of surgeons working in 75 countries, revealed significant differences in *S*. *aureus* colonization between the regions, with the lowest colonization rates in surgeons working in Africa and the highest in South America. A prevalence of 21% for *S*. *aureus* from 79 North American surgeons, is lower than that described by Schwarzkopf *et al*. in a smaller cohort of surgeons [43]; although whether this is reflective of a downwards trend in the US is difficult to determine at this point. Previous studies have shown increased prevalence of *S*. *aureus* in white males [44, 45]. Although we did not record the ethnicity of participants, we could identify a low *S*. *aureus* colonization in our African population. The tendency for increased *S*. *aureus* colonization rates in certain regions is likely a multifactorial one involving a significant genetic component [44, 46–48], although this requires further investigation.

The antibiotic resistance profile of these bacteria is an important issue, with the CDC identifying MRSA as a serious threat to public health [4]. MRSA prevalence in our survey averaged 2%, although significant regional variations were observed. The highest rates of MRSA colonization were found in Asia, Central America and Africa. There was no MRSA detected within our population of 79 surgeons working in North America, and a low MRSA rate in 657 surgeons working in Europe. The absence of MRSA in North American surgeons was unexpected. However, given the high positive culture yield, the data strongly indicates a low prevalence in North America. The tendency for higher MRSA rates in Asia and Africa may be expected when one considers the high previously reported MRSA rates (42–73%) in HCWs in regional small-scale studies [49, 50]. MRSA colonization in HCWs has also been described for Norway and Cape Verde (0%), USA, (2%), São Tomé and Príncipe (4.0%) and Iran (5.3%) [37, 39, 40, 42, 51]. Exceedingly high MRSA colonization rates in HCW of 41.9% in Uganda and over 70% in Saudi Arabia [49, 50]. No significant effect of age, gender or type of hospital was found to be significant in relation to MRSA carriage in our study, although our dataset was significantly gender biased. Nevertheless, age has previously been shown to be a factor in *S*. *aureus* colonization [52, 53], which could not be repeated within our study.

MRSA colonization rates in the general population are quite consistently below 2%, as shown for large studies in Northern Europe (<1%), with higher levels described in isolated reports from Central/Western-European countries, the US, New Zealand and Australia (6–22%) and higher still in Southern European countries as well as in parts of the US, Asia and Africa (28–63%) [34, 36, 54, 55]. A trend for increasing MRSA prevalence in the US healthcare system has been described in the past, with in two US National Health and Nutrition Examination Surveys (NHANES) showing a significant increase of MRSA colonization within non-institutionalized patients from 0.8% to 1.5% between 2001 and 2004 [56]. Our MRSA prevalence of 0% in North American surgeons reflects a significant reduction in comparison with the earlier New York Study [43]. The annual report 2013 of the European Antimicrobial Resistance Surveillance Network (EARS-Net) has revealed generally lower MRSA percentages in Northern Europe and higher rates in south and southeastern countries [57]. This study could not confirm this trend since MRSA colonization was detected in just four European participants. In addition to a trend of lowering MRSA prevalence across European countries, the improved antimicrobial stewardship practices may also have a side effect of reducing HCW colonization in this region.

Twenty-two percent of participating surgeons received antibiotic treatment in the six months prior to the study. This did not cause an increase in culture negative results in our study, and experimental studies have shown only a short-term reduction in bacterial load secondary to antibiotic usage [58]. We found that antibiotic therapy simultaneously reduced the rate of MSSA colonization, and increased the rate of MRSA and MRCoNS colonization compared with the remaining surgeons. This has not been shown in a surgical population previously, but does mirror patient studies, wherein MRSA prevalence was increased after exposure to antibiotics as revealed in a meta-analysis from 2008 [59]. Therefore, mirroring the calls to rationalize patient antibiotic usage, protocols to ensure antibiotic stewardship for HCWs is a topic also requiring some attention.

In this study, we additionally analyzed MRCoNS carriage, which literature tells us can reach 75% in invasive infections in the hospital setting [10]. Scattered studies report MRCoNS nasal colonization rates of outpatients are much lower, ranging from 11% to 31% [60]. Scant data is available on MRCoNS in surgeon populations. We identified an overall MRCoNS colonization rate of 21.4%, which is quite high, although within the limits of the general population. Geographic differences were again apparent, with North America and Europe again being low, and Asia and Africa with the highest prevalence. Within Europe, methicillin resistance in CoNS ranged from a low of 6.1% in Northern Europe to a high of 25.0% in Eastern Europe. The reasons for a high MRCoNS incidence in Asia and Africa are poorly understood, but a crossover in parameters that lead to high MRSA incidence may be considered likely. With an increasing awareness of MRCoNS as a pathogen and as a source of mobile antibiotic resistance genes, the risk of antibiotic resistance gene transfer to *S*. *aureus* should not be underestimated [25, 61]. Some resistant CoNS displayed resistance against rifampicin, daptomycin and linezolid. In infections caused by methicillin resistant staphylococci, these antibiotics may represent the only possible treatment options [62]. Colonization with such strains is a cause for concern and indicates further studies may be required to monitor the scale of colonization with bacteria resistant to important antibiotics.

One of the most discussed topics when it comes to HCW colonization with resistant bacteria is always going to be the impact upon the HCW themselves, their employers and the patients being treated. A proposed solution for colonization with *S*. *aureus* and MRSA has been the development of decolonization protocols [63, 64]. Decolonization with MRCoNS is not described and, as universal commensals, decolonization of CoNS is not practical. Decolonization has been shown to be more efficacious in surgical departments, as results have been less promising for other specialties [63, 64], and thus surgeons may represent a special case for more regular screening.

There are a number of limitations to this study. In particular, this study monitors colonization at a single time-point, although colonization is known to be variable over time and it has to be differentiated between persistent and intermitted carriers [2]. Repeat sampling of this diverse and large population of surgeons would require either re-swabbing at each local hospital or recruiting the proportion that return to the same event 12 months later. In any case, storing surgeon data was not permitted according to our IRB approval, rendering repeat sampling impossible. Another potential limitation may be that the detection rate of staphylococci may have been increased if a DNA based evaluation were performed (e.g. PCR). Since the swabs were not moistened, and there was no pre-enrichment step before plating on blood agar, prevalence of *S*. *aureus*, MRSA and MRCoNS may, at least in theory, be underestimated. The choice of swab type (MASTASWAB^TM^, Mast Group Ltd., Italy) may influence the MRSA carrier status as shown by Warnke *et al*. in an *in vitro* model and may have led to diagnosing false negative MRSA carrier status [65]. Similarly, the swabs were taken at an altitude of 1,400 meters in the Swiss Alps, although previous findings indicate this should not be an issue [45, 66]. Ultimately, our culture positive rate of 95.3% clearly indicates the swab and sample handling techniques were highly successful. An unavoidable limitation of the study is the unequal distribution of participants from each region. The study had a high enrollment rate, and the study profile largely reflected the overall participant profile in all aspects (age, gender, region of work). Finally, a more detailed and expanded questionnaire could have addressed further interesting research questions such as details on treatment with antibiotics, previous decolonization, hospital prescribing practices, length of employment at current institution. However, we minimized the time required for enrollment in an effort to maximize recruitment and so further questions were not asked.

The data presented in this study indicates surgeons are broadly equivalent to the general population in terms of nasal colonization with resistant bacteria. As stated in the recent report (2014) of the World Health Organization on antimicrobial resistance, antibiotic prescribing and infection control practices vary throughout the world [67]. These may affect the significant geographic variation of nasal colonization with resistant strains. Importantly, the surgical profession cares for a vulnerable population, and further studies would be required to elucidate the impact of increased surveillance on the relationship between surgeons and their own health, but also on the role this may play in nosocomial infections.

## Conclusions

This study shows that the rate of nasal colonization with *S*. *aureus* (26%) and MRSA (2%) in surgeons is similar to the general population. Significant geographic variation was observed, which may be at least partially accounted for by varying antimicrobial stewardship practices in the different regions. Concerted efforts within the USA and Europe to achieve greater antibiotic prescribing restraint, may partially explain the reduced colonization with resistant bacteria in these regions.
